# Patterns and predictive factors of unhealthy practice among mothers during pregnancy, childbirth, postnatal and newborn care in Southern Ethiopia: a community based cross-sectional study

**DOI:** 10.1186/s13104-019-4631-3

**Published:** 2019-09-18

**Authors:** Getinet Kassahun, Negash Wakgari, Ribka Abrham

**Affiliations:** 1 Department of Midwifery, College of Medicine and Health Science, Hawassa University, Hawassa, Ethiopia; 2 Minilik Hospital, Addis Ababa, Ethiopia

**Keywords:** Unhealthy practice, Patterns, Magnitude, Predictors, Pregnancy, Childbirth, Postpartum, Newborn

## Abstract

**Objective:**

The aim of this study was to assess the magnitude, patterns and predictive factors of unhealthy practice among mothers during pregnancy, childbirth, postnatal and newborn care in Southern Ethiopia.

**Results:**

Among the total participants, 29.0% mothers performed at least one unhealthy practice during pregnancy, childbirth, postnatal period and newborn care. This study identified the following harmful practices such as food prohibition (53.2%), home delivery (41.5%), discarding colostrum (18.6%), application of substance on the cord stump (12.1%), delayed breast feeding (28.4%), prelacteal feeding (43.0%) and early bathing (49.3%). Being grand multiparous (AOR = 2.528, 95% CI 1.037–6.166), being illiterate (AOR = 7.611, 95% CI 2.375–24.396) and lack of awareness on the effect of unhealthy practice (OR = 4.673, 95% CI 1.163–18.774) were independent predictors of outcome variable.

## Introduction

Globally, about 287,000 women die from the complication of pregnancy, childbirth and postpartum [1]. About 1 million and 2.5 million children died on the first day of life and in the first 4 weeks of life, respectively [2]. In Ethiopia, maternal mortality rate stands at 497 per 100,000 live-births [3]. Moreover, the neonatal, infant and under-5 mortality rate accounts for 29, 48, and 67 deaths per 1000 live births, respectively [4].

Most of neonatal deaths are easily preventable [5]. Maternal deaths can be also prevented by the early recognition of complications and use of appropriate emergency referral systems as well as by providing timely and adequate care [6]. However, delayed recognition of illness and sociocultural factors are the most common blockades to seeking care. Still, it was the major challenge to seek and reach timely accessing life-saving emergency obstetric care [7].

Neonatal health and survival can be improved by universal application of essential newborn care following delivery. These practices include cleanliness, delaying of first bath, thermal protection, breathing initiation, early and exclusive breastfeeding, eye care, immunization, and treatment of neonatal illness [8]. However, unhealthy practices which may cause infections, hypothermia, anemia and hypoglycemia and thus increase the risk neonatal diseases [9].

The time during pregnancy, childbirth and neonatal period is culturally very important time and existing behaviours are strong adherence to traditional practices. In addition to the practice of essential newborn care, successful behaviour change strategies should be also required [9, 10]. In Ethiopia, there are different culture, religion and socio-economic backgrounds. Understanding cultural practice in study area is a crucial to improve maternal and neonatal outcomes as identifying knowledge gaps and harmful behaviour would help to minimize the harmful practices. Hence, the aim of this study was to assess the magnitude, patterns and predictive factors of unhealthy practice among mothers during pregnancy, childbirth, postpartum and newborn care in Southern Ethiopia.

## Main text

### Methods

#### Study design

A community based cross-sectional study design.

#### Study area and period

The study was conducted at Wonago town in Southern region of Ethiopia. It is located 380 km far from capital city of Ethiopia (Addis Ababa). The study was conducted from March 10 to April 9, 2018.

#### Source population

All mothers gave birth within 3 years prior proceeding the study period in Wonago town during the study period.

#### Study population

All sampled mothers who gave birth within 3 years prior proceeding the study period were included in the study. But, mothers who were seriously ill during data collection were excluded.

#### Sample size determination and sampling techniques

The sample size of 297 mothers was determined by using a single population proportion formula with confidence interval 95%, margin of error 5%, prevalence of unhealthy practice 25.6% [11] and adding 10% non-response rate.

Based on Wonago town health administrative report, a total of 2015 mothers gave birth within 3 years prior proceeding the study period. Then, households (HHs) of the identified eligible population will be registered to create a sampling frame. Lastly, 297 mothers from Wonago Town were selected using a simple random sampling technique. When sampled mother was absent from their home on the data collection day, the re-visited the household was arranged.

#### Variable measurement

Unhealthy practice: is the cultural practice of reproductive health during pregnancy, childbirth, postnatal period and newborn care which bring a higher risk of morbidity and mortality for women and newborn.

Untrained traditional birth attendant: is a birth attendant without receiving training on the basic mother–child care.

Early bathing: is the practice of bathing a newborn before 24 h of birth.

Delay breastfeeding: is the practice of breast feeding a newborn after 1 h of birth.

Colostrum avoidance: is the practice of avoiding the first yellowish milk feeding (highly nutritious and protective the newborn from diseases).

Prelacteal feeding: is the practice of feeding babies with culture specific foods immediately after birth other than breast milk.

Neonates/Newborn: is a baby in the first 4 weeks of life.

Grand multipara: mother who give birth five and more.

#### Study variables

The dependent variable was unhealthy practice. Any form of unhealthy practices was dichotomized as yes and no. Yes if the mother reported any one or more any form of unhealthy practices or No if any form of unhealthy practices were not reported. Then, Yes was computed and coded as ‘1’, while No was computed and coded as ‘0’ for logistic regression analysis.

The independent variables were socio-demographic factors (maternal age, ethnicity, religion, residence, educational status, occupation, marital status, average monthly income and number of live birth), knowing about the effect of unhealthy practice, place of birth and birth attendant were also assessed as explanatory variables.

#### Data collection procedure and quality assurance

Data was collected using a structured interviewer-administered face to face interview. Questionnaire was initially prepared in English and translated into local languages (Amharic and Gedeogna) by reviewing different previous articles and back-translated to the English language by three native speakers to check its consistency. Data quality was managed by trained BSc. Midwifery data collectors and pre-test on 5% of sample size. Collected data was checked by daily supervision.

#### Data processing and analysis procedures

The collected and coded data were entered into EpiData version 3.1 and then exported to SPSS version 21.0 statistical package for analysis. Firstly, univariable analysis was used to describe the continuous and categorical variables (frequency, percentage, mean and standard deviation). Then, bivariable logistic regression was conducted to assess crude association between outcome and independent variables. Those significant variables at p-value < .25 during bivariable analyses were included in the multivariable analyses. Lastly, multivariable logistic regression was conducted to assess adjusted association by controlling the potential confounders. Adjusted odds ratio (AOR) was used to determine the strength of association between dependent and independent variables. Variables with p value < .05 at 95% confidence interval (CI) were declared as statistically significant in multivariable logistic regression analysis.

### Results

#### Socio‑demographic characteristics of the study participants

A total of 297 mothers were enrolled in the study with a response rate of 97.6%. The mean (± SD) age of mothers in the study was 28 ± 6 years (ranged from 15 to 45 years). Majority (72.8%), (84.5%), (78.3%) and (73.1%) of the mothers were married, Christian, illiterate and rural. 141 (48.6%), 68 (23.4%), 61 (21.4%) and 19 (6.6%) of participants were Gedeo, Oromo, Guraghe and Amhara by ethnic group, respectively. Almost half (48.6%) of the mothers were farm produce respectively. One hundred twenty-nine (44.5%) of the mothers had a monthly income below 500 birr (Table 1).

**Table 1:** Socio-demographic characteristic of participants

Variables	Frequency	Percentage
Age (in years)		
15–24	6	2.1
25–34	167	57.6
≥ 35	117	40.3
Marital status		
Married	211	72.8
Separate/single	79	27.2
Religion		
Christian	245	84.5
Muslim	45	15.5
Educational status		
Literate	63	21.7
Illiterate	227	78.3
Occupation		
Farm produce	153	52.8
Business/self employed	84	29.0
Employed/salaried	53	18.3
Residence		
Urban	78	26.9
Rural	212	73.1
Monthly income		
< 500ETB	129	44.5
501–999ETB	119	41.0
≥ 1000ETB	42	14.5
Number of live birth		
1–4	144	49.7
≥ 5	146	50.3

Key ETB = Ethiopian birr (1US$ = 27ETB)

#### Awareness regarding unhealthy practice

Fifty (17.2%) of mothers had awareness on the effect of unhealthy practice. From all participants, 10 (3.4%), 13 (4.5%), 12 (4.1%), 9 (3.1%) and 6 (2.0%) mentioned malnutrition, bleeding, anemia, infections and obstructed labor, respectively.

#### Magnitude and pattern of unhealthy practice

In this study, eighty-four (29%) of participants performed at least one unhealthy practice during pregnancy, childbirth, postnatal and newborn care.

Regarding pregnancy, 46 (15.0%) of participants consumed craved foods and 154 (53.2%) of them had food prohibition. Concerning birth place and attendant, 120 (41.5%) of participants were assisted by untrained TBA at home and 80 (27.7%) of mothers applied abdominal massage with butter to facilitate labor.

Regarding umbilical cord care, thirteen (4.5%) of respondents used unclean blade to cut umbilical cord and nineteen (6.6%) of them used unclean thread to tie umbilical cord. 22 (7.6%) of participants untied umbilical cord with thread whereas twelve (4.0%) of them tied umbilical cord with their leg. 35 (12.1%) of respondents applied substance (dry cow dung or ashes or powder) on the cord stump.

Concerning breast feeding, 54 (18.6%) mothers discarded the colostrum (first yellowish milk) and 125 (43.0%) of them gave pre lacteal feeding (butter, honey, sugar and water). 208 (71.6%), 59 (20.4%) and 23 (8.0%) of mothers gave breast feeding the first time were within 1 h, within 24 h and after 24 h of delivery, respectively.

Regarding initial time of bathing, 84 (29.0%), 59 (20.3%) and 147 (50.7%) of mothers washed their babies within 1 h, within 24 h and after 24 h of birth, respectively. 179 (62%) of mothers kept their babies from sunlight exposure (Table 2).

**Table 2:** Frequency of unhealthy practice during pregnancy, childbirth, postnatal and neonatal period in southern Ethiopia, 2018 (N = 290)

Variables	Frequency	Percentage
Abdominal massage with butter		
No	210	72.3
Yes	80	27.7
Nutritional prohibition during pregnancy		
No	136	46.8
Yes	154	53.2
Consumption of craved foods		
No	244	85.0
Yes	46	15.0
Place of birth		
Health facility	170	58.5
Home	120	41.5
Tying of the cord to her leg		
No	278	96.0
Yes	12	4.0
Instrument used to cut the umbilical cord		
Unclean blade	13	4.5
Clean blade	277	95.5
Material used to tie cord		
Unclean	19	6.6
Clean	271	93.4
The substance on cord stump		
No	253	87.9
Yes	35	12.1
Colostrum feeding		
No	54	18.6
Yes	236	81.4
Pre lacteal feeding		
No	165	57.0
Yes	125	43.0
Keeping babies out of exposure to sun		
No	179	62.0
Yes	111	38.0
Time to start breastfeeding		
Within 1 h	208	71.6
Within 24 h	59	20.4
After 24 h	23	8.0

#### Predictors of unhealthy practice

Bivariable and multivariable logistic regression analyses were done to assess the association between the selected variables and unhealthy practice. During adjusted binary logistic regression analysis, three explanatory variables were statistically significant associated with unhealthy practice. Grand multipara mothers were 2.528 times more likely to perform unhealthy practice than those who had 1–4 live birth (AOR = 2.528, 95% CI 1.037–6.166). The odd of unhealthy practice was 7.611 among illiterate mothers as compared to literate mothers (AOR = 7.611, 95% CI 2.375–24.396). The odd of unhealthy practice was also 4.673 among mothers who have no information on the effect of unhealthy practice as compared to their counterpart (OR = 4.673, 95% CI 1.163–18.774) (Table 3).

**Table 3:** Predictive factors of unhealthy practice during pregnancy, childbirth, postnatal and neonatal period in southern Ethiopia, 2018 (N = 290)

Category	Unhealthy practice		Crude OR (95% CI)	Adjusted OR (95% CI)
	No	Yes		
Knowing the effect of unhealthy practice				
No	60	180	2.769 (1.479–5.184)*	4.673 (1.163–18.774)*
Yes	24	26	1	1
Number of live birth				
≥ 5	56	90	2.578 (1.516–4.382)*	2.528 (1.037–6.166)*
1–4	28	116	1	1
Religion				
Muslim	19	26	2.024 (1.050–3.900)*	.783 (.177–3.467)
Christian	65	180	1	1
Residence				
Rural	70	142	2.254 (1.182–4.296)*	.496 (.196–1.253)
Urban	14	64	1	1
Educational status				
Illiterate	79	148	6.192 (2.386–16.066)*	7.611 (2.375–24.396)*
Literate	5	58	1	1
Birth attendant				
Untrained attendant	43	77	1.757 (1.052–2.933)*	.692 (.312–1.536)
Trained attendant	41	129	1	1

*CI* Confidence interval, *COR* crude odds ratio, *AOR* adjusted odds ratio

*Statistically significant (p < .05), ‘1’ reference category

### Discussion

From the total study subjects during study period, 29% of mothers performed at least one unhealthy practice during pregnancy, childbirth, postnatal and neonatal period. The frequency of home delivery in our study was 41.5%. This finding was greater than reported in other studies [12]. However, it was lower than that reported in other studies [13, 14]. The prevalence of substance application (dry cow dung or ashes or powder) on stump of umbilicus was 12.1%. This finding is less than with other study [15, 16]. It is harmful practice as it could be the risk of infections to the newborn.

The frequency of food prohibition during pregnancy in our study was 53.2%. This finding is greater than other studies [12, 17]. It could be the risk of anemia and malnutrition to the newborn and the mother. The frequency of colostrum avoidance immediately after birth was 18.6%. It was greater than other studies [15, 18]. This finding is less than with other study [19]. It is unhealthy practice as the first milk feeding is highly nutritious, digested and protective the newborn from diseases. The prevalence of prelacteal feeding (butter, honey, sugar and water) soon after delivery was 43%. This finding consistent with other study [16]. But, it was greater than that reported in other studies [15, 20]. Feeding other than breast milk soon after birth increases the risk for infection as well as less nutritive to the newborn. In this study, the prevalence of early breast feeding in our study was 71.6%. This finding is greater than with other study [15, 21].

Being grand multipara was more likely associated with unhealthy practice in our study. The reason could be elder mothers accepted as cultural practice from the past generation. Education status was more likely associated with unhealthy practice. This finding is observed in other study [12, 15]. This may be due to having education supports mothers to have information on the healthy practice and access maternal and child care services. Lack of awareness on effect of unhealthy practice was more likely associated with outcome variable.

#### Clinical implications of the findings

This study might have an implication maternal and child health care; by identifying the gaps it supports to reduce maternal and neonatal morbidity and mortality and to improve quality of reproductive health care.

### Conclusion

The magnitude of unhealthy practice was high in Southern Ethiopia. This study identified the following unhealthy practices such as food prohibition, home delivery, discarding colostrum, unhygienic cord cutting, delayed breastfeeding, prelacteal feeding and early bathing. Grand multipara, literate and lack of awareness on the effect of unhealthy practice were predictive factors of outcome variable.

Community education and behaviour change messages are recommended to minimize unhealthy care during pregnancy, childbirth, postnatal and neonatal period.

## Limitation of the study

First, the nature of cross-sectional study design could not establish the cause and effect relationship. Secondly, there is no assessing of qualitative findings for possible triangulation. Lastly, there is possible recall bias among mothers relating to events happening in the past.

## Data Availability

There is no remaining data and materials; all information is documented in the main manuscript.

## Abbreviations

AOR: adjusted odd ratio
BSC: Bachelor of Science
CI: confidence interval
ETB: Ethiopian Birr
OR: odd ratio
SD: standard deviation
SPSS: statistical package for social science
TBA: traditional birth attendant
